# Comparison between bacteremia caused by carbapenem resistant *Acinetobacter baumannii* and *Acinetobacter nosocomialis*

**DOI:** 10.1186/1471-2334-13-311

**Published:** 2013-07-10

**Authors:** Ya-Sung Yang, Yi-Tzu Lee, Wen-Chiuan Tsai, Shu-Chen Kuo, Jun-Ren Sun, Chin-Hsuan Yang, Te-Li Chen, Jung-Chung Lin, Chang-Phone Fung, Feng-Yee Chang

**Affiliations:** 1 Division of Infectious Diseases and Tropical Medicine, Department of Internal Medicine, Tri-Service General Hospital, National Defense Medical Center, Taipei, Taiwan; 2 Institute of Clinical Medicine, School of Medicine, National Yang-Ming University, Linong Street, Taipei 112, Taiwan; 3 Emergency Department, Taipei Veterans General Hospital, Taipei, Taiwan; 4 Department of Pathology, Tri-Service General Hospital, National Defense Medical Center, Taipei, Taiwan; 5 National Institute of Infectious Diseases and Vaccinology, National Health Research Institutes, Miaoli County, Taiwan; 6 Clinical Microbiology Laboratory Division of Clinical Pathology, Tri-Service General Hospital, Taipei, Taiwan; 7 Centers for Disease Control, Department of Health, Taipei, Taiwan

**Keywords:** *Acinetobacter*, Bacteremia, Carbapenem resistant, Risk factor, Mortality

## Abstract

**Background:**

It is unknown whether there are differences between bacteremia caused by carbapenem resistant *Acinetobacter baumannii* (CRAB) and carbapenem resistant *Acinetobacter nosocomialis* (CRAN). This study aims to investigate the differences, especially in clinical outcomes, between patients with bacteremia caused by CRAB or CRAN.

**Methods:**

This is a 9-year retrospective study comparing the clinical manifestations, antimicrobial susceptibilities, and clinical outcomes of 71 patients with CRAB bacteremia and 64 patients with CRAN bacteremia.

**Results:**

Patients with CRAB were more likely to have hematologic malignancies and presented with more shock episodes than those with CRAN. CRAB isolates were more resistant to various classes of antimicrobials except colistin, and therefore the patients with CRAB bacteremia were more likely to receive inappropriate antimicrobial therapies. The 14-day mortality was significantly higher in patients with CRAB (40.8% vs. 14.1%; *p* = 0.001), and in this study, acquisition of CRAB was identified as an independent risk factor for mortality (odds ratio = 4.003; 95% confidence interval = 1.566-10.231; *p* = 0.004).

**Conclusions:**

CRAB and CRAN bacteremia are different in clinical characteristics, antimicrobial susceptibilities, and mortality rates. Genomic species identification should be performed in the study of carbapenem resistant *Acinetobacters* to better delineate the role of different species.

## Background

*Acinetobacter* species have emerged as important pathogens causing nosocomial infections [1]. Carbapenem resistant *Acinetobacter* spp. are now increasingly encountered worldwide [2,3], especially in the Asia-Pacific region [4-6]. The three most clinically relevant *Acinetobacter* species, *Acinetobacter baumannii*, *Acinetobacter nosocomialis* (formerly *Acinetobacter* genomic species 13TU), and *Acinetobacter pittii* (formerly *Acinetobacter* genomic species 3), cannot be differentiated by phenotypic tests used in clinical microbiological laboratories and are grouped as the *A*. *baumannii* (Ab) group [1]. Though the prevalence of *Acinetobacter* species in the Ab group vary in different geographic areas and institutions, *A*. *baumannii* and *A*.*nosocomialis* are commonly isolated clinical *Acinetobacter* species worldwide [7-12] and accounting for more than 80% of clinical infections caused by the Ab group in Taiwan [10,13,14].

Compared to bacteremia caused by *A*. *nosocomialis*, bacteremia caused by *A*. *baumannii* is associated with worse clinical outcomes [10,12,13,15]. The higher mortality in patients with *A*. *baumannii* might be attributed to a higher pathogenicity of *A*. *baumannii *[10,15]. However, it is unknown whether the more severe clinical outcomes of *A*. *baumannii* bacteremia compared to *A*. *nosocomialis* bacteremia will be the same if both the pathogens are carbapenem resistant, as it has been shown that the virulence of *A*. *baumannii* resistant to certain drugs is impaired [16,17]. In this study, we aim to compare the clinical characteristics, antimicrobial susceptibilities of the bacterial isolates, and especially the clinical outcomes of patients with bacteremia caused by carbapenem resistant *A*. *baumannii* (CRAB) and *A*. *nosocomialis* (CRAN).

## Methods

### Study population

The study was conducted at the Taipei Veterans General Hospital (T-VGH) during a nine-year period from January 2000 to December 2008. T-VGH is a 2900-bed tertiary-care teaching hospital located in Taipei, Taiwan. Data analyses were performed at the Tri-Service General Hospital (TSGH), National Defense Medical Center in Taipei, Taiwan.

Charts were reviewed from all patients with at least one positive blood culture for *A*. *baumannii* or *A*. *nosocomialis* who had symptoms and signs of infection. Only the first blood culture from patients with two or more positive blood cultures was included. The source of infection was determined as recommended in Centers of Disease Control guidelines [18,19]. Bacteremia cases without a definite identified source were defined as primary bacteremia. Patients under 18 years of age and those with incomplete medical records were excluded. The protocol was approved by the T-VGH and TSGH Institutional Review Board (approval number: 2011-10-012IC and 2-101-05-074, respectively) with a waiver for informed consent.

### Microbiological studies

The presumptive identification of the isolates to the level of the Ab group was performed with the API ID 32 GN system (bioMérieux, Marcy l’Etoile, France) or Vitek 2 system (bioMérieux, Marcy l’Etoile, France). A multiplex-PCR method was used to identify *A*. *baumannii* to the genomic species level [20]. Isolates identified as non-*A*. *baumannii* species of *Acinetobacter* were identified to the genomic species level by 16S–23S ribosomal DNA intergenic spacer sequence analysis [21]. Antimicrobial susceptibilities were determined by the agar dilution method according to the Clinical Laboratory Standards Institute (CLSI) [22]. Multidrug resistance was defined as resistance to three or more of the following classes of antimicrobial agents: anti-pseudomonal cephalosporins, anti-pseudomonal carbapenems, ampicillin/sulbactam, fluoroquinolones, and aminoglycosides [1].

### Molecular typing

The clonal relationships of the clinical isolates were determined by pulsed-field gel electrophoresis (PFGE) [23]. Twenty randomly selected isolates from each *Acinetobacter* species were performed. PFGE of *Apa*I-digested genomic DNA was performed using the Bio-Rad CHEF-Mapper apparatus (Bio-Rad Laboratories, Hercules, CA, USA). Cluster analysis was performed using BioNumerics version 5.0 (Applied Maths, Sint-Martens-Latem, Belgium) and the unweighted pair-group method with arithmetic averages (UPGMA). The Dice correlation coefficient was used with a tolerance of 1% in order to analyze any similarities between banding patterns. Isolates showing more than three DNA fragment differences and a similarity of <80% following dendrogram analysis were considered to represent different pulsotypes.

### Data collection

Medical records were reviewed to extract clinical information, including demographic characteristics, underlying diseases, duration of stay in an intensive care unit (ICU), hospital stay, time of receipt, dose and route of administration of individual antimicrobials, and the presence of a ventilator, central venous catheters, a nasogastric tube, or a foley catheter at the time of onset of bacteremia. Immunosuppressive therapy was defined as receipt of cytotoxic agents within 6 weeks, corticosteroids at a dosage equivalent to or higher than 10 mg of prednisolone daily for more than 5 days within 4 weeks, or other immunosuppressive agents within 2 weeks prior to the onset of bacteremia. Neutropenia was defined as an absolute neutrophil count <0.5×10^9^ neutrophils/L. Recent surgery was defined as operations performed within 4 weeks prior to the onset of bacteremia. Chronic kidney disease was defined as an estimated glomerular filtration rate <60 mL/min/1.73 m^2^. Shock was defined as hypotension (systolic blood pressure [SBP] <90 mmHg, mean arterial pressure <70mmHg, or a SBP decrease >40 mmHg) with evidence of end organ dysfunction [24]. Polymicrobial bacteremia was defined as isolation of one or more microorganisms other than *A*. *baumannii* or *A*. *nosocomialis* from blood during the same bacteremic episode. The severity of illness was evaluated using the Acute Physiology and Chronic Health Evaluation II (APACHE II) score [25] within 24 hours prior to bacteremia onset.

Appropriate antimicrobial therapy was defined as administration of at least one antimicrobial agent, to which the causative pathogen was susceptible, within 48 hours after the onset of bacteremia, with an approved route and dosage for end organ(s) function. Antimicrobial therapy that did not meet this definition was considered inappropriate. Monotherapy with an aminoglycoside was not considered an appropriate therapy. The primary outcome measure was all-cause 14-day mortality following the onset of CRAB or CRAN bacteremia.

### Statistical analysis

To assess differences, the chi-square test with Yate’s correction or Fisher’s exact test was used to compare the discrete variables, while the Student’s *t*-test or Mann–Whitney rank sum test was used to analyze continuous variables. Logistic regression models were used to explore independent risk factors for 14-day mortality. Univariate analyses were performed separately for each of the risk factor variables to ascertain the odds ratio (OR) and 95% confidence interval (CI). All biologically plausible variables with a *p* value of ≤0.10 in the univariate analysis exhibited by at least 10% of the patients were considered for inclusion in the logistic regression model for the multivariate analysis. A backward selection process was utilized. Time to mortality was analyzed using Kaplan-Meier survival analysis and long-rank test. A *p* value <0.05 was considered statistically significant. All the analyses were processed with Statistical Package for the Social Sciences (SPSS) software version 18.0 (SPSS, Chicago, IL, USA).

## Results

During the study period, 801 patients were found to have at least one episode of Ab group bacteremia. Among them, 185 (23.1%) patients with polymicrobial bacteremia were excluded from the study. Following genomic species analysis, 259 (42.0%) and 294 (47.7%) patients were identified as having bacteremia caused by *A*. *baumannii* and *A*. *nosocomialis*, respectively. The final population that met the entry criteria for this study consisted of 71 patients with CRAB bacteremia and 64 patients with CRAN bacteremia. A total of 43 patients were excluded due to young age (13 patients <18 years) and incomplete medical records (30 patients).

Among the 20 randomly selected CRAB and CRAN isolates, the analysis of PFGE showed that there were 7 and 14 pulsotypes, respectively (data not shown).

The demographic and clinical characteristics of the patients included in the study are summarized in Table 1. The most common source of CRAB and CRAN bacteremia was the respiratory tract (66.2% and 70.3%, respectively). Patients with CRAB had hematologic malignancies more frequently than those with CRAN (12.7% vs. 1.6%, *p* = 0.019). Though APACHE II scores at the onset of bacteremia were similar in both groups, shock was more frequently present in patients with CRAB.

**Table 1:** Demographic and clinical characteristics of patients with bacteremia caused by carbapenem resistant *Acinetobacter baumannii *and carbapenem resistant Acinetobacter nosocomialis*

**Demographic or characteristic**	***A. baumannii *(n=71)**	***A. nosocomialis *(n=64)**	***p *value**
	**n (% or interquartile range)**		
Age in years	75(61.0–82.0)	76(62.0–81.0)	0.78
Gender, male	54(76.1)	46(71.9)	0.7
Acquired in ICU	60(84.5)	54(84.4)	1
Days of hospitalization prior to culture	22.5(9.5–39.5)	25(8.0–34.0)	0.26
Source			
Respiratory tract	47(66.2)	45(70.3)	0.71
Intravenous device	4(5.6)	7(10.9)	0.35
Intra-abdomen	6(8.5)	3(4.7)	0.5
Wound	2(2.8)	1(1.6)	1
Primary bacteremia	12(16.9)	8(12.5)	0.32
Comorbidity			
Coronary artery disease	7(9.9)	11(17.2)	0.31
Congestive heart failure	5(7.0)	11(15.6)	0.11
Cerebral vascular disease	13(18.3)	17(26.6)	0.3
Hypertension	22(31.0)	25(39.1)	0.37
COPD	14(19.7)	20(31.2)	0.17
Alcoholism	7(9.9)	1(1.6)	0.07
Liver cirrhosis	3(4.2)	3(4.7)	1
Diabetes mellitus	20(28.2)	22(34.4)	0.46
Collagen vascular disease	4(5.6)	4(6.2)	1
Usage of immunosuppressants	23(32.4)	15(23.4)	0.26
Neutropenia	2(2.8)	1(1.6)	1
Malignancy	27(38.0)	24(37.5)	1
Hematologic malignancy	9(12.7)	1(1.6)	0.019
Solid malignancy	20(28.2)	23(35.9)	0.36
Recent surgery	26(36.6)	29(45.3)	0.38
Trauma	2(2.8)	2(3.1)	1
Procedure†			
Abdominal drain	7(9.9)	9(14.1)	0.6
Central venous catheter	49(69.0)	47(73.4)	0.7
Foley catheter	53(74.6)	44(48.8)	0.57
Hemodialysis	9(12.7)	8(12.5)	1
Thoracic drain	1(1.4)	4(6.2)	0.19
Total parental nutrition	9(12.7)	7(10.9)	0.8
Endotracheal tube or tracheostomy	53(74.6)	53(82.8)	0.3
Ventilator	49(69.0)	45(70.3)	1
Arterial line	22(31.0)	12(18.8)	0.12
Nasogastric tube	61(85.9)	51(79.7)	0.37
APACHE II score†	26(19.0–34.0)	26(18.3–31.8)	0.1
Shock	29(40.8)	10(15.6)	0.003
Appropriate antimicrobial therapy	9(12.7)	22(34.4)	0.004
Mortality	43(60.6)	21(32.8)	0.002
14-day mortality	29(40.8)	9(14.1)	0.001
28-day mortality	34(47.9)	13(20.3)	0.001
Time to mortality, day	7(2.0–26.0)	21(9.5–68.5)	0.022

*Data are median value (interquartile range) for continuous variables and number of cases (%) for categorical variables. †Within 24 hours prior to bacteremia onset. *APACHE II* Acute Physiologic and Chronic Health Evaluation II, *COPD* chronic obstructive pulmonary disease, *ICU* intensive care units.

The antimicrobial susceptibilities of the clinical isolates of CRAB and CRAN are shown in Table 2. CRAB exhibited significantly higher rates of resistance to all antimicrobials tested, except colistin and piperacillin/tazobactam. Only one *A*. *baumannii* isolate was resistant to colistin (1.4%), while in contrast, 45.3% (29/64) of the *A*. *nosocomialis* isolates were resistant to colistin (*p*<0.001). Clinical isolates of CRAB exhibited multidrug resistance more frequently than CRAN (100% vs. 75%, *p*<0.001). Patients with CRAB more frequently received inappropriate antimicrobial therapies than those with CRAN (Table 1).

**Table 2:** **Comparison of antimicrobial susceptibilities of clinical isolates of carbapenem resistant *Acinetobacter baumannii *and carbapenem resistant *Acinetobacter nosocomialis***

	**Resistance, n (%)**		
**Antimicrobial agent**	***A. baumannii *(n=71)**	***A. nosocomialis *(n=64)**	***p *value**
Amikacin	68(95.8)	30(46.9)	<0.001
Gentamicin	68(95.8)	54(84.4)	0.038
Ceftazidime	69(97.2)	14(21.9)	<0.001
Cefepime or cefpirome	49(69.0)	13(20.3)	<0.001
Ampicillin/sulbactam	43(60.6)	27(42.2)	0.039
Piperacillin/tazobactam	56(78.9)	42(65.6)	0.12
Ciprofloxacin	69(97.2)	4(6.2)	<0.001
Colistin	1(1.4)	29(45.3)	<0.001
Tigecycline	6(8.2)	0(0)	0.03

The antimicrobials used, the APACHE II score, appropriateness of antimicrobial use, and patient outcomes are shown in Table 3. Due to the limited number of cases, differences in illness severity, and appropriateness of therapy in each therapy group, it was difficult to evaluate the effect of different antimicrobial therapies on patients with CRAB and CRAN bacteremia. Overall, the mortality rate of CRAB was higher than CRAN in different antimicrobial therapy groups. For example, in the carbapenem therapy groups, in which the illness of severity was comparable between both groups, the 14-day mortality rates in CRAB and CRAN groups were 41.2% vs. 14.3%, respectively. In the sulbactam therapy group, although the illness severity was lower in the CRAB group, the 14-day mortality rate was still higher (45.5 vs. 0%).

**Table 3:** Antimicrobials used for the patients with carbapenem resistant *Acinetobacter baumannii *and *Acinetobacter nosocomialis *bacteremia*

**Main agents used**	**No. of patients**	**APACHE II score**	**No. (%) of patients**	
		**Median (interquartile range)**	**Appropriate antimicrobial therapy**	**14–day mortality**
Anti-pseudomonal penicillins^a^				
CRAB	12	31.5(22.8–39.0)	2(16.7)	6(50.0)
CRAN	8	24(19.0–33.0)	7(87.5)	2(25.0)
Anti-pseudomonal cephalosporins^b^				
CRAB	14	26(22.8–34.0)	2(14.3)	4(28.6)
CRAN	10	26(19.3–30.0)	7(70.0)	1(10.0)
Anti-pseudomonal fluoroquinolones^c^				
CRAB	5	30(21.0–36.0)	1(20.0)	2(40.0)
CRAN	2	37.5(37.0–38.0)	2(100.0)	0(0)
Anti-pseudomonal carbapenems^d^				
CRAB	17	32(24.0–38.5)	0(0)	7(41.2)
CRAN	14	30.5(23.0–33.3)	0(0)	2(14.3)
Ampicillin/sulbactam or sulbactam				
CRAB	11	17(15.0–33.0)	4(36.4)	5(45.5)
CRAN	8	24(18.0–27.5)	3(37.5)	0(0)
Non-antipseudomonal β-lactamases^e^				
CRAB	6	17.5(15.3–25.3)	0(0)	1(16.7)
CRAN	9	18(15.5–24.0)	3(33.3)	1(11.1)
Miscellaneous				
CRAB	6	25(17.8–32.0)	0(0)	2(33.3)
CRAN	13	27(16.0–35.5)	0(0)	3(23.1)

*Data are median value (interquartile range) for continuous variables and number of cases (%) for categorical variables. ^a^Including piperacillin, piperacillin/tazobactam, and ticarcillin/clavulanate. ^b^Including cefoperazone, ceftazidime, cefepime, and cefpirome. ^c^Including ciprofloxacin and levofloxacin. ^d^Including imipenem and meropenem. ^e^Including penicillin, amoxicillin/clavulanate, cefazolin, cefuroxime, cefotaxime, cefmetazole, and flomoxef. *APACHE II* Acute Physiologic and Chronic Health Evaluation II, *CRAB* carbapenem resistant *A*. *baumannii*, *CRAN* carbapenem resistant *A*. *nosocomialis*.

**Figure 1:**
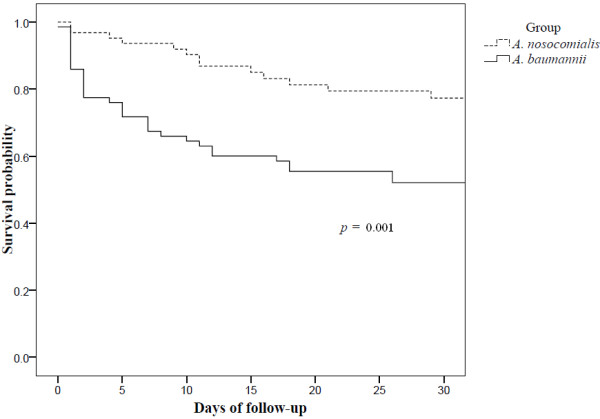
**Kaplan**-**Meier survival curves for patients with carbapenem resistant *Acinetobacter baumannii *bacteremia and carbapenem resistant *Acinetobacter nosocomialis *bacteremia.**

The mortality rates by 14 and 28 days after bacteremia onset were significantly higher in patients with bacteremia caused by CRAB than CRAN (*p* = 0.001). The 14-day mortality for CRAB was 40.8% (29/71) compared with 14.1% (9/64) for CRAN. The average time from admission to death was significantly shorter in CRAB than CRAN(16.7 days vs. 39.4 days, *p* = 0.022). Kaplan-Meier survival curves reflected the early occurrence of death within a few days following bacteremia for patients infected with CRAB. This was followed by higher rates of death in the CRAB group than the CRAN group (*p* = 0.001, by log-rank test) (Figure 1).

Factors associated with 14-day mortality are summarized in Table 4. Bacteremia with CRAB, higher APACHE II score, use of immunosuppressants and shock were univariable risk factors for mortality. Following logistic regression analysis, *A*. *baumannii* itself was found to be an independent risk factor for mortality among all patients (odds ratio = 4.003; 95% confidence interval = 1.566-10.231; *p* = 0.004). The usage of immunosuppressants was also an independent risk factor for mortality.

**Table 4:** **Factors associated with 14**-**day mortality in patients with carbapenem resistant *Acinetobacter baumannii *and *Acinetobacter nosocomialis *bacteremia**

**Demographic and characteristics**	**Univariate analysis**		**Multivariate analysis**	
	**Odds ratio**	***p *value**	**Odds ratio**	***p *value**
	**(95% CI)**		**(95% CI)**	
*A*. *baumannii*	4.220(1.806–9.861)	0.001	4.003(1.566–10.231)	0.004
APACHE II score*	1.165(1.098–1.237)	<0.001		
Score ≤25	1.127(0.965–1.317)	0.131		
Score >25	1.062(0.986–1.144)	0.113		
Score ≤35	1.086(1.012–1.166)	0.022		
Score >35	1.056(0.895–1.247)	0.113		
Cerebral vascular disease	0.222(0.063–0.783)	0.019	0.194(0.048–0.789)	0.022
Usage of immunosuppressants	4.105(1.827–9.227)	0.001	3.921(1.516–10.143)	0.005
Appropriate antimicrobial therapy	0.381(0.144–1.004)	0.051		
Recent surgery	0.190(0.073–0.494)	0.001	0.222(0.079–0.628)	0.005
Shock	2.760(1.244–6.122)	0.013		

*Every one increase in APACHE II score is accompanied by an increase of 1.165 in the odds ratio. *APACHE II* Acute Physiologic and Chronic Health Evaluation II, *CI* Confidence interval.

## Discussion

This study clearly demonstrates significant differences in the risk factors, antimicrobial resistances, and especially clinical outcomes between bacteremia caused by CRAB and CRAN. Patients infected with CRAB were more likely to have hematologic malignancies and a greater frequency of shock. Even though both pathogens were carbapenem resistant, CRAB exhibited resistance to more antimicrobial agents. CRAB bacteremia was also associated with more frequent instances of inappropriate antimicrobial use, as well as more rapid and higher mortality rates. Among the patients with CRAB and CRAN bacteremia, acquisition of CRAB was found to be an independent factor for 14-day mortality.

Compared with CRAN, CRAB had higher resistance rates toward most commonly used antimicrobial agents, and all CRAB isolates were multidrug resistant. This resistance is responsible for the increased chance of receiving an inappropriate antimicrobial therapy in patients infected with these microorganisms. On the contrary, there were still some therapeutic options available for the treatment of CRAN bloodstream infections. A higher rate of colistin resistance in *A*. *nosocomialis* than *A*. *baumannii* was also observed in previous reports [14,26,27], where the resistance of colistin in *A*. *nosocomialis* was about 20%. In this study, it was even higher in CRAN isolates (45.3%).

The higher mortality of patients infected with CRAB compared to CRAN may be attributed to unfavorable underlying diseases, an increased likelihood of inappropriate antimicrobial therapy in the former group, and possibly higher pathogenicity of *A*. *baumannii*[10,13]. According to our findings, there were more shock episodes in patients with CRAB than with CRAN of similar disease severity. Furthermore, CRAB was identified as an independent risk factor for mortality after adjustment for other risk factors for mortality, including underlying diseases, severity of illness, and appropriateness of antimicrobial therapy. This result indicates a possible higher pathogenicity of CRAB compared to CRAN. Because of the diversity of the clonality of CRAB and CRAN isolates, we believe that the result is due to species but not strain effect. Genomic species identification is therefore important to better delineate the role of different species of carbapenem resistant *Acinetobacters*[21,28].

The prevalence of *Acinetobacter* species in the Ab group can vary in different geographic areas and institutions. For example, *A*. *pittii* was found to be the predominant species accounting for clinical infections in Germany during 2005-2009 [11]. Although *A*. *baumannii* and *A*. *nosocomialis* account for more than 80% of clinical infections caused by the Ab group, their ratio differs among centers in Taiwan [10,13-15]. Three centers demonstrated a >2:1 relative ratio of *A*. *baumannii* to *A*. *nosocomialis*[10,13,15], however, the ratio has consistently been around 1:1 in our center in recent years [26,29].

This study is subject to several limitations regularly found in retrospective studies. Several confounding factors, especially the antimicrobials used during the whole treatment course, could not be well controlled in both groups. However, it is not likely that certain antimicrobial regimens that might affect clinical outcomes were used more often in either group as the primary physicians were unaware of the genomic species.

## Conclusions

CRAB and CRAN bacteremia are different in clinical characteristics, antimicrobial susceptibilities, and mortality rates. Genomic species identification should be performed in the study of carbapenem resistant *Acinetobacters* to better delineate the role of different species.

## Abbreviations

A. baumannii: *Acinetobacter baumannii*; Ab group: *A*. *baumannii* group; A. nosocomialis: *Acinetobacter nosocomialis*; APACHE II: Acute Physiology and Chronic Health Evaluation II; CLSI: Clinical Laboratory Standards Institute; COPD: Chronic obstructive pulmonary disease; CRAB: Carbapenem resistant *A*. *baumannii*; CRAN: Carbapenem resistant *A*. *nosocomialis*; DNA: Deoxyribonucleic acid; ICU: Intensive care unit; PCR: Polymerase chain reaction; PFGE: Pulsed-field gel electrophoresis; SBP: Systolic blood pressure; SPSS: Statistical Package for the Social Sciences; TSGH: Tri-Service General Hospital; T-VGH: Taipei Veterans General Hospital.

## Competing interests

Te-Li Chen is a medical advisor for TTY Biopharm. Other authors declare that they have no competing interests.

## Authors’ contributions

YSY, YTL, and TLC participated in the study design, analysis of data, and writing of the manuscript. JRS and SCK participated in the data collection, while WCT and CHY participated in the data analysis. JCL, CPF, and FYC revised the manuscript with important intellectual contributions. All authors read and approved the final manuscript.

## Pre-publication history

The pre-publication history for this paper can be accessed here:

http://www.biomedcentral.com/1471-2334/13/311/prepub

## References

[B1] Peleg AY Seifert H Paterson DL *Acinetobacter baumannii*: emergence of a successful pathogen Clin Microbiol Rev 2008 21 3 538 582 10.1128/CMR.00058-07 18625687 PMC2493088

[B2] Higgins PG Dammhayn C Hackel M Seifert H Global spread of carbapenem-resistant *Acinetobacter baumannii* J Antimicrob Chemother 2010 65 2 233 238 10.1093/jac/dkp428 19996144

[B3] Kempf M Rolain JM Emergence of resistance to carbapenems in *Acinetobacter baumannii* in Europe: clinical impact and therapeutic options Int J Antimicrob Agents 2012 39 2 105 114 10.1016/j.ijantimicag.2011.10.004 22113193

[B4] Kuo SC Chang SC Wang HY Lai JF Chen PC Shiau YR Huang IW Lauderdale TL Hospitals T Emergence of extensively drug-resistant *Acinetobacter baumannii* complex over 10 years: nationwide data from the Taiwan Surveillance of Antimicrobial Resistance (TSAR) program BMC Infect Dis 2012 12 200 10.1186/1471-2334-12-200 22929085 PMC3462144

[B5] Lin YC Sheng WH Chen YC Chang SC Hsia KC Li SY Differences in carbapenem resistance genes among *Acinetobacter baumannii*, *Acinetobacter* genospecies 3 and *Acinetobacter* genospecies 13TU in Taiwan Int J Antimicrob Agents 2010 35 5 439 443 10.1016/j.ijantimicag.2009.11.020 20106635

[B6] Xiao YH Giske CG Wei ZQ Shen P Heddini A Li LJ Epidemiology and characteristics of antimicrobial resistance in China Drug resistance updates: reviews and commentaries in antimicrobial and anticancer chemotherapy 2011 14 4–5 236 250 21807550 10.1016/j.drup.2011.07.001

[B7] Boo TW Walsh F Crowley B Molecular characterization of carbapenem-resistant *Acinetobacter* species in an Irish university hospital: predominance of *Acinetobacter* genomic species 3 J Med Microbiol 2009 58 Pt 2 209 216 19141738 10.1099/jmm.0.004911-0

[B8] Karah N Haldorsen B Hegstad K Simonsen GS Sundsfjord A Samuelsen O Norwegian study group of A: species identification and molecular characterization of Acinetobacter spp. blood culture isolates from Norway J Antimicrob Chemother 2011 66 4 738 744 10.1093/jac/dkq521 21393175

[B9] Turton JF Shah J Ozongwu C Pike R Incidence of *Acinetobacter* species other than *A*. *baumannii* among clinical isolates of *Acinetobacter*: evidence for emerging species J Clin Microbiol 2010 48 4 1445 1449 10.1128/JCM.02467-09 20181894 PMC2849580

[B10] Chuang YC Sheng WH Li SY Lin YC Wang JT Chen YC Chang SC Influence of genospecies of *Acinetobacter baumannii* complex on clinical outcomes of patients with acinetobacter bacteremia Clin Infect Dis 2011 52 3 352 360 10.1093/cid/ciq154 21193494

[B11] Schleicher X Higgins PG Wisplinghoff H Korber-Irrgang B Kresken M Seifert H Molecular epidemiology of *Acinetobacter baumannii* and *Acinetobacter nosocomialis* in Germany over a 5-year period (2005-2009) Clin Microbiol Infectin press 10.1111/1469-0691.12026 23034071

[B12] Wisplinghoff H Paulus T Lugenheim M Stefanik D Higgins PG Edmond MB Wenzel RP Seifert H Nosocomial bloodstream infections due to *Acinetobacter baumannii*, *Acinetobacter pittii* and *Acinetobacter nosocomialis* in the United States J Infect 2012 64 3 282 290 10.1016/j.jinf.2011.12.008 22209744

[B13] Lee YC Huang YT Tan CK Kuo YW Liao CH Lee PI Hsueh PR ***Acinetobacter baumannii *****and *****Acinetobacter*** genospecies 13TU and 3 bacteraemia: comparison of clinical features, prognostic factors and outcomes J Antimicrob Chemother 2011 66 8 1839 1846 10.1093/jac/dkr200 21653602

[B14] Lee YT Huang LY Chiang DH Chen CP Chen TL Wang FD Fung CP Siu LK Cho WL Differences in phenotypic and genotypic characteristics among imipenem-non-susceptible *Acinetobacter* isolates belonging to different genomic species in Taiwan Int J Antimicrob Agents 2009 34 6 580 584 10.1016/j.ijantimicag.2009.06.027 19733035

[B15] Lee NY Chang TC Wu CJ Chang CM Lee HC Chen PL Lee CC Ko NY Ko WC Clinical manifestations, antimicrobial therapy, and prognostic factors of monomicrobial *Acinetobacter baumannii* complex bacteremia J Infect 2010 61 3 219 227 10.1016/j.jinf.2010.07.002 20624424

[B16] Lopez-Rojas R Dominguez-Herrera J McConnell MJ Docobo-Perez F Smani Y Fernandez-Reyes M Rivas L Pachon J Impaired virulence and in vivo fitness of colistin-resistant *Acinetobacter baumannii* J Infect Dis 2011 203 4 545 548 10.1093/infdis/jiq086 21216865 PMC3071218

[B17] Smani Y Lopez-Rojas R Dominguez-Herrera J Docobo-Perez F Marti S Vila J Pachon J In vitro and in vivo reduced fitness and virulence in ciprofloxacin-resistant *Acinetobacter baumannii* Clin Microbiol Infect 2012 18 1 E1 4 10.1111/j.1469-0691.2011.03695.x 22084991

[B18] Garner JS Jarvis WR Emori TG Horan TC Hughes JM CDC definitions for nosocomial infections, 1988 Am J Infect Control 1988 16 3 128 140 10.1016/0196-6553(88)90053-3 2841893

[B19] Horan TC Gaynes RP Mayhall CG, Baltimore MD Surveillance of nosocomial infections Hospital epidemiology and infection control 2004 3 Lippincott Williams: Wilkins 1659 1702

[B20] Chen TL Siu LK Wu RC Shaio MF Huang LY Fung CP Lee CM Cho WL Comparison of one-tube multiplex PCR, automated ribotyping and intergenic spacer (ITS) sequencing for rapid identification of *Acinetobacter baumannii* Clin Microbiol Infect 2007 13 8 801 806 10.1111/j.1469-0691.2007.01744.x 17488329

[B21] Chang HC Wei YF Dijkshoorn L Vaneechoutte M Tang CT Chang TC Species-level identification of isolates of the *Acinetobacter calcoaceticus*-*Acinetobacter baumannii* complex by sequence analysis of the 16S-23S rRNA gene spacer region J Clin Microbiol 2005 43 4 1632 1639 10.1128/JCM.43.4.1632-1639.2005 15814977 PMC1081347

[B22] Clinical and Laboratory Standards Institute (CLSI) Performance standards for antimicrobial susceptibility testing: Twenty-first informational supplement 2011 Wayne, PA: CLSI document M100-S21

[B23] Seifert H Dolzani L Bressan R Van der Reijden T Van Strijen B Stefanik D Heersma H Dijkshoorn L Standardization and interlaboratory reproducibility assessment of pulsed-field gel electrophoresis-generated fingerprints of *Acinetobacter baumannii* J Clin Microbiol 2005 43 9 4328 4335 10.1128/JCM.43.9.4328-4335.2005 16145073 PMC1234071

[B24] Dellinger RP Levy MM Carlet JM Bion J Parker MM Jaeschke R Reinhart K Angus DC Brun-Buisson C Beale R Surviving Sepsis Campaign: international guidelines for management of severe sepsis and septic shock: 2008 Crit Care Med 2008 36 1 296 327 10.1097/01.CCM.0000298158.12101.41 18158437

[B25] Knaus WA Draper EA Wagner DP Zimmerman JE APACHE II: a severity of disease classification system Crit Care Med 1985 13 10 818 829 10.1097/00003246-198510000-00009 3928249

[B26] Liang-Yu C Kuo SC Liu CY Luo BS Huang LJ Lee YT Chen CP Chen TL Fung CP Difference in imipenem, meropenem, sulbactam, and colistin nonsusceptibility trends among three phenotypically undifferentiated *Acinetobacter baumannii* complex in a medical center in Taiwan, 1997-2007 J Microbiol Immunol Infect 2011 44 5 358 363 10.1016/j.jmii.2011.01.032 21524973

[B27] Nemec A Dijkshoorn L Variations in colistin susceptibility among different species of the genus *Acinetobacter* J Antimicrob Chemother 2010 65 2 367 369 10.1093/jac/dkp440 20008049

[B28] Gundi VA Dijkshoorn L Burignat S Raoult D La Scola B Validation of partial rpoB gene sequence analysis for the identification of clinically important and emerging *Acinetobacter*species species Microbiology 2009 155 Pt 7 2333 2341 19389786 10.1099/mic.0.026054-0

[B29] Lee YT Kuo SC Yang SP Lin YT Chiang DH Tseng FC Chen TL Fung CP Bacteremic nosocomial pneumonia caused by *Acinetobacter baumannii* and *Acinetobacter nosocomialis*: a single or two distinct clinical entities? Clin Microbiol Infect 2013 19 7 640 645 10.1111/j.1469-0691.2012.03988.x 22967204

